# A Comparative Analysis of the Efficacy of Bacterial Lysate versus Antibiotic Therapy in the Treatment of Experimental Peri-Implantitis in Rats

**DOI:** 10.3390/microorganisms12081537

**Published:** 2024-07-27

**Authors:** Diana Larisa Ancuţa, Diana Mihaela Alexandru, Cătălin Ţucureanu, Cristin Coman

**Affiliations:** 1 Cantacuzino National Medical Military Institute for Research and Development, 050096 Bucharest, Romania; diana.larisa.ancuta@gmail.com (D.L.A.); catalintucureanu@gmail.com (C.Ţ.); 2 Faculty of Veterinary Medicine, University of Agronomic Sciences and Veterinary Medicine, 050097 Bucharest, Romania; 3 Center of Excellence in Translational Medicine, Fundeni Clinical Institute, 022328 Bucharest, Romania

**Keywords:** peri-implantitis treatment, bacterial lysate, antibiotic therapy, rodent model, immunological response

## Abstract

Peri-implantitis (PI) is a current concern whose understanding and resolution are ongoing. We aimed to evaluate in vivo a new treatment with antibacterial properties, based on bacterial lysates obtained from the strains of *Aggregatibacter actinomycetemcomitans*, *Streptococcus oralis*, and *Fusobacterium nucleatum.* This research was conducted on 30 rats with PI which were divided into three groups and treated with antibiotic and anti-inflammatory (AAi) drugs, bacterial lysates (BLs), and saline (C), respectively. The monitoring period included the clinical and paraclinical examination where hematological, immunological, imaging, and histopathological analysis were performed. No particular clinical signs were observed, but the radiological examination showed the loss of all implants in group C, in contrast to group BL which had the highest survival rate of devices. White cells showed a decrease from the PI period, as did the immunological analysis. Only IL-6 showed an increase in the AAi and BL groups. Histopathologically, the C group presented a high degree of bone destruction, and in the BL group, many attenuated inflammatory phenomena appeared compared to the AAi animals. Bacterial lysates have similar effects to antibiotic-based therapeutic regimens for PI, and their future use may help to improve the current therapeutic management of the disease.

## 1. Introduction

Dental implants have recently become the most convenient solution for replacing lost teeth. They greatly improve the quality of life although they are very prone to bacterial infection [1]. Peri-implantitis starts with the inflammation of the connective tissue around the implant, then progresses rapidly and without following a standard course, until the loss of bone support. Clinical manifestations are varied and often not similar among patients so that signs of inflammation, the formation of pockets of different sizes on probing, bleeding, and/or oozing, and circular bone resorption phenomena may be observed [1].

The etiological factors of peri-implantitis are multiple, and the associated risk factors are closely related to chronic periodontal disease (PD) and include poor oral hygiene, smoking, systemic diseases (diabetes), and genetic factors. Peri-implantitis and periodontal disease share similar pathophysiological aspects although some studies claim significant differences between them [2]. Clinically and radiologically, PI and PD share common features, but the anatomical–histological environment, microbiome, and immune aspects draw a clear demarcation between the two conditions. Thus, the lesions observed in PI are significantly more pronounced than those in PD, as the immune response is generated [3]. In PI, granulation tissue in the peri-implant site is heavily impregnated with proinflammatory cytokines compared to tissues in periodontal sites [4]. Some findings indicate other factors responsible for PI, and among them, we find residual submucosal cement from restorations, the defective position of implants in the bone, or the lack of peri-implant keratinized membrane [1]. Interestingly, peri-implantitis is not a condition that occurs immediately after fitting but, on average, after about 5 years [3], which is sufficient time for peri-implant plaque accumulation. 

One of many threats to the stability of an implant is this bacterial plaque [5] that develops and acts on the implant similar to a periodontal situation [6]. The peri-implant microbiota compared to the periodontal microbiota is worse in terms of quality, but quantitatively, it is higher for some bacterial genera [7]. The adhesion and colonization of bacteria on the surface of an implant ultimately result in the formation of biofilm, and in the process of composition, bacteria contribute gradually, which has led to their classification into early and late colonizers [8]. Biofilm developed on the surface of dental implants is a major problem because in more than 50% of cases, it causes irreversible tissue destruction [9]. The first bacterial strains that adhere to the surface of an implant belong to the genus *Streptococcus* (*Streptococcus oralis, Streptococcus mitis*), and later, anaerobic strains of *Porphyromonas gingivalis* (with black pigment) or *Actinobacillus actinomycetemcomitansitans* adhere and even migrate into the subgingival space [10]. For bacterial species to cooperate in the formation of peri-implant biofilm, Kolenbrander et al. found that the presence of *Fusobacterium nucleatum* (*F. nucleatum*), which can coaggregate both streptococci and *Actinomyces* spp., is required, in addition to *Porphyromonas* spp. or *Prevotella* spp. [11]. *F. nucleatum* appears as an incriminating microbial agent as early as the mucositis stage, and the fact that it is found on the surface of implants in advanced stages of disease makes its major role in the progression of peri-implantitis more than obvious [12]. The bacterial composition of implant-associated biofilms encompasses a wide variety of species, in addition to those mentioned above, including *Treponema denticola*, *Tarnerella forsythia*, *Veionella* spp., etc. [13].

The development of these biofilms must be prevented, especially in areas where effective oral hygiene cannot be ensured. Cases have been detailed in which the migration of biofilm bacteria has endangered the health of implants [14], and the development of effective therapeutic methods and techniques in treating PI is essential to eliminate this risk. The current therapeutic management for peri-implantitis is derived from the strategies used to treat periodontitis, but they often have unpredictable results [15]. Biofilms are usually resistant to the action of the host immune system and even to antibiotic treatments [16], so the only way to resolve peri-implantitis remains the surgical approach complemented using disinfectants and antibiotics [6]. Due to the excessive use of disinfectants and biocides, antibiotic-based therapeutic strategies have suffered, and therefore, immediate alternative solutions are needed to act against the pathogens responsible for peri-implantitis [17]. These refer to materials incorporated into the implant structure or administered preventively or curatively. Three types of adjuvant measures have been found for the non-surgical therapy of PI in recent years: local antimicrobial measures (minocycline microspheres, chlorhexidine chips, or a metronidazole + amoxicillin gel), systemic measures (amoxicillin + metronidazole, either metronidazole alone), and probiotics (*Lactobacillus reuteri* strains) [18,19,20,21]. Only local treatment has reduced effects, but systemic treatment significantly improves probing depth reduction and/or bleeding on probing [22].

The complexity of peri-implantitis and its therapy is difficult to understand, so further studies involving animal models are needed. In recent years, several protocols have been tried to induce PI in rodents and test different treatment regimens, allowing researchers to conduct studies much more efficiently, with lower costs and shorter healing time [23].

Rodents have several advantages for assessing microbial and host responses. Studies using these animal models have demonstrated the induction of disease by placing ligatures around implants, resulting in the accumulation of bacterial biofilm [24], but these studies have focused on the etiopathogenesis or therapy of peri-implantitis and less on quantifying biofilm following the application of a novel therapeutic regimen [13]. Potential strategies to combat peri-implantitis are still being explored, and a remedy that interferes with the adhesion and colonization of bacteria in the implant is needed. In this regard, experimental studies contribute to the progress of novel therapies, even if the results obtained from in vivo tests have been translated into the clinical sphere in only 20% of cases [25]. The purpose of preclinical trials is to test the safety and efficacy of new therapies; before they are tested in humans and until a solution is found that will facilitate the path from the laboratory to marketing to patients, a product must be developed that has outstanding efficacy comparable to current therapies. In this light, in our study, we aimed to evaluate in vivo a new treatment with antibacterial properties, intended for PI, based on bacterial lysates obtained from the strains of *Aggregatibacter actinomycetemcomitans* (*A. actinomycetemcomitans*), *Streptococcus oralis* (*S. oralis*), and *F. nucleatum*. This research was performed on a rat model in which PI was induced by oral contamination with the same bacteria from which the experimental treatment was performed, and the null hypothesis was based on the similar response of the lysates to antibiotic therapy in fighting the bacterial agents involved. Through this hypothesis, the ability of bacterial lysates to act on the bacterial strains involved in the induction of PI by inhibiting their growth and colonization on the implant surface and deep in the peri-implant tissues can be demonstrated. The immune response triggered by the administration of bacterial lysates offers the advantage of avoiding the administration of antibiotics, and the antibiotic-like effects make bacterial lysates a high-potential alternative for the treatment of PI.

## 2. Materials and Methods

### 2.1. Ethics Statement

This study was conducted in accordance with Law 43/2014 and Directive 2010/63/EU on the protection of animals used for scientific purposes. All steps of the experiment were approved by the Ethics Committee of the Faculty of Veterinary Medicine Bucharest, Romania (no 25/15 June 2022), and by the Sanitary, Veterinary and Food Safety Directorate Bucharest, Romania, no 27/29 August 2022, with entry number 8224/10 August 2022. This manuscript follows the guidelines provided in “The ARRIVE guidelines 2.0: author checklist—The ARRIVE Essential 10” [26], the animal experiments taking place in the Preclinical Testing Unit of the Cantacuzino National Medical-Military Institute for Research and Development, Bucharest, Romania (CI), a unit authorized for the use of animals for scientific purposes.

### 2.2. The Cultural Conditions of the Bacterial Strains Used for PI Induction

*A. actinomycetemcomitans* (ATCC 29522), *S. oralis* (DSM 20627), and *F. nucleatum* (ATCC 25586) from CI’s bacterial strain collection in the form of aliquots stored in glycerol were handled and grown under anaerobic conditions (80%N_2_, 10%CO_2_, and 10%H_2_). The culture medium used to grow the three strains was Schaedler Anaerobe Broth (Oxoid, Thermo Scientific, Newton Drive, Carlsbad, CA, USA), and 24 h incubated cultures were used on each day of oral inoculation. As in a previous study [27], the dose used was 0.6 mL inoculum (0.2 mL of each bacterium), 5 days/week, for 6 weeks, and the concentration of each bacterium stable for oral contamination was 10^9^ CFU/mL determined by the nephelometric method (Densitometer McFarland Biosan DEN-1, Riga, Lithuania). 

### 2.3. Bacterial Lysate Production Method

The 24 h cultures of *A. actinomycetemcomitans*, *S. oralis*, and *F. nucleatum*, the same cultures that were also used for PI induction, were inactivated in a water bath for 1 h. To verify the efficiency of the inactivation, the resulting suspensions were inoculated onto Brain Heart Infusion (BHI), Sabouraud, and Tyoglycolate (Oxoid, Thermo Scientific, Newton Drive, Carlsbad, CA, USA) culture media and then incubated for 14 days at 37 °C. During this time, the tubes with control media were checked daily for any change in turbidity that might indicate bacterial growth. After inactivation control, the bacterial wall lysis process followed. The method by which this was carried out was a mechanical one involving the ultrasonication (Ultrasonic CD-4801, Shenzhen, China) of the inactivated suspensions in successive 8 min cycles for 1 h. The effect of the lysates obtained was tested in vitro by cytotoxicity tests (MTT method), the efficacy was verified by contacting different volumes of lysate with live bacteria [28], and we concluded that at least a double dose of lysate inhibited bacterial growth on both solid and liquid culture media. In in vivo testing, we decided to administer rats with PI at a dose of 1.2 mL by oral lavage for 10 days.

### 2.4. Animals

This study included 30 male, 20-week-old Wistar rats with an average weight of 400 g at the start of the experiment. They were supplied by CI’s Specified Pathogen-Free Animal Facility and then housed in CI’s Preclinical Testing Unit experimental space. Throughout this study, rats were housed under conventional conditions, including nesting material, with unlimited access to food (Granulated Combined Feed—NCG, produced by CI’s Combined Feed Plant) and water ad libitum. 

The general health of all animals was checked daily. Implant-specific health status was documented every other day during the experiment, starting after the first surgery, and body weight was monitored every 14 days.

Exclusion criteria were determined prior to the start of the study and included the following conditions: a weight loss of 20% or more at any time during the experiment, which would also result in the immediate euthanasia of the animal or death before the final day. 

### 2.5. Study Design

**Figure 1:**
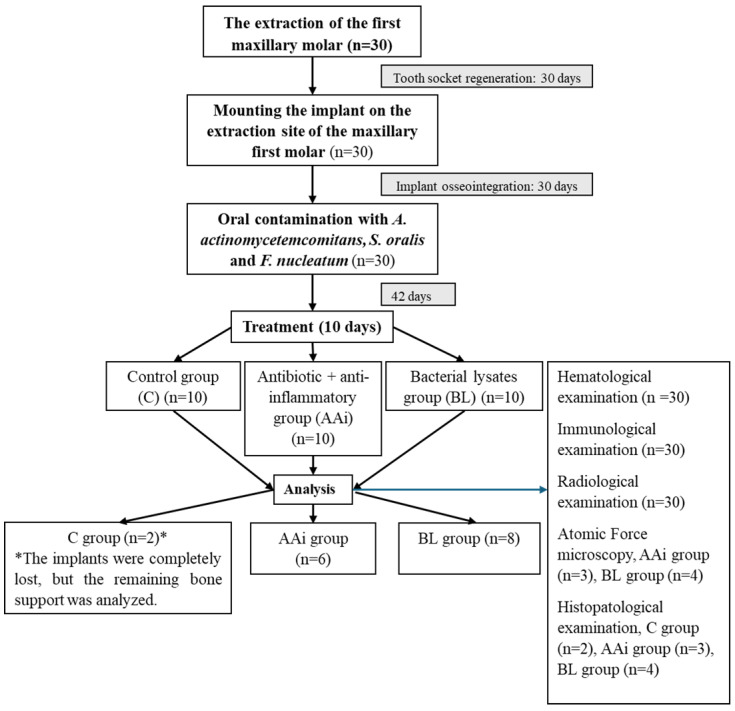
A diagram and consort flow chart of the experimental procedures in this study.

This study was carried out in 4 major steps, as shown in Figure 1, which involved the extraction of the maxillary first molar, the fixation of the implant on the extracted molar site, and oral contamination with *A. actinomycetemcomitans*, *S. oralis*, and *F. nucleatum*, previously described when creating the PI model [27]. After the completion of the bacterial inoculation period (day 102), we allocated the animals into 3 groups (10 rats/group) to be treated as follows: saline-treated group (C), antibiotic- and anti-inflammatory-treated group (AAi), and bacterial lysate-treated group (BL). As in the case of oral contamination, treatments were also administered by lavage and gavage for 10 days.

### 2.6. Peri-Implantitis Rat Model Protocol

At the onset of the experiment (day 0), all animals were weighed, and blood samples were taken from the retro-orbital sinus for hematological and immunological analysis. Depending on individual weight, the rats were anesthetized with Medetomidine (0.5 mg/kg, Biotur, Alexandria, Romania) and Ketamine (0.5 mg/kg, Farmavet, Bucharest, Romania) and then underwent the dental extraction procedure as described in a previous article [29]. The period we allowed for the restoration of bone support before implant mounting was 30 days. The appearance of the implant bed was checked by clinical and especially radiological examination, performed on day 30. The implant mounting procedure included the creation of a cavity (using an X-Cube implant motor, Saeshin America, Irvine, CA, USA) with a diameter of 1 mm and 4 mm depth into which the titanium device of the same dimensions was inserted. To protect the implant from masticatory forces, the gingiva was sutured with 4/0 multifilament resorbable thread (Serafit, Serag Weissner, Naila, Germany). Postoperatively, the animals were treated with antibiotics and anti-inflammatories for 3 days (Enrofloxacin 10%, 2.5 mg/kg, Farmavet, Bucharest, Romania, and Ketoprofen, 3 mg/kg, Dopharma, Giroda, Timiș, Romania). The period required for the osseointegration of the implant, as in the previous step, was also 30 days, and the fixation of the implant in the bone was also checked radiologically on day 60.

The third phase of this study aimed at enriching the microbiota of rats with bacteria responsible for PI in humans (*A. actinomycetemcomitans*, *S. oralis*, and *F. nucleatum*, whose processing is described in Section 2.2) for 6 weeks; then, after the specific signs of PI were confirmed (day 102), we proceeded to the treatment, as shown in Figure 1, when animals of group AAi received 40 mg/kg amoxicillin with clavulanic acid (Amoksiklav, 600 mg/42.9 mg/5 mL, powder for oral suspension, Sandoz, Bucharest, Romania) and acetaminophen (Tis Paracetamol for children, 120 mg/5 mL, 100 mL, Tis Farmaceutic, Bucharest, Romania). The BL group was treated with 1.2 mL lysate in which 0.4 mL lysate of *A. actinomycetemcomitans*, *S. oralis*, and *F. nucleatum* was present, and the control group was treated with 0.6 mL saline.

At the end of 10 days of treatment, the animals were euthanized by anesthetic overdose, and implant jaw samples were collected for histopathological analysis.

### 2.7. The Monitoring of Animals and Examinations throughout This Study

The assessment of PI induction was performed by daily clinical examination, monitoring the appearance of the gingiva, bleeding or oozing on implant percussion, device mobility, or loss. Moreover, even after the treatments were instituted, the clinical signs were followed, especially in the BL group, for any signs of intolerance.

The hematological examination performed on day 0, day 60, day 102, and day 112 followed the analysis of the parameters responding to the bacterial infection (lymphocytes, neutrophils, monocytes) and to the treatment applied. For this, we collected retro-orbital blood in EDTA tubes (KIMA Vacutest, Arzergrande, Italy) which we analyzed with the Idexx Procyte 5 diff analyzer.

The immunoassay included an analysis of the proinflammatory cytokines interleukin 1b (IL-1b), interleukin 6 (IL-6), and Tumor Necrosis Factor α (TNF-α). For this, the LXSARM-3 kit (R&D Systems Inc., Minneapolis, MN, USA) was acquired, and duplicate blood samples collected from the Rat Luminex Discovery Assay were run on Lithium Heparin vacutainers.

Radiological examination was performed on days 30, 60, and 102 on the IVIS Lumina XRMS Werner ROEDL (PerkinElmer, Traiskirchen, Austria) to check post-extraction bone regeneration and the integration of the implant into the bone after oral contamination and after the application of treatments.

On the final day of the experiment, parts of the implant jaw samples were collected in containers of 4% formalin, fixed for 1 week, and then further processed for a fluorescent and atomic force microscopy evaluation of potential bacterial biofilms on the surface. Implants were carefully extracted by performing a proximal incision in the bone (around 1 mm from the implant) using a circular saw and then removing the surrounding bone with forceps. Control samples were prepared in parallel by incubating implants for 72 h in culture media and an inoculation of *A. actinomycetemcomitans*, *S. oralis*, and *F. nucleatum*. Implant samples were washed in phosphate buffer saline (PBS) and stained for 30 min with 5 μg/mL DAPI (4′,6-Diamidino-2-phenylindole dihydrochloride) and 1 μg/mL eosin (Sigma-Aldrich, Merck Romania SRL, Bucharest, Romania) in PBS at room temperature. Fluorescent micrographs covering the whole implant were acquired with a 4× objective on a Nikon Ti microscope equipped with a DS-Qi2 monochrome camera, using DAPI and TxRed filters (Nikon BioImaging Labs—Leiden, The Netherlands). Acquisition was controlled with Micro-Manager [30], and individual tiles were fused using the Grid/Collection stitching plugin [31] in Fiji [32].

Implants were washed extensively with water and dried in atmospheric air for 48 h before being mounted in plast support putty (PELCO—Agar Scientific, Stansted, UK) for atomic force microscopy analyses. Atomic force microscopy (AFM) was performed on an alpha300 RAS microscope (WITec GmbH, Ulm, Germany) using 240AC-NG silicone cantilevers (Opus, Sofia, Bulgaria), with a nominal frequency of 70 kHz and a nominal spring constant of 2 N/m. All scans were performed in air, in AC mode, at a scan rate of 0.5–1 Hz. Multiple 10 × 10 µm scans were randomly acquired on different areas of the implants (more than 20 scans each for the in vivo implants and 5 scans for the in vitro controls). Scans were processed in Gwyddion [33], rows were aligned by median and mean plane subtraction, and topography and amplitude images were exported on fixed Z scales for all samples (0–500 nm for topography and 0–300 mV for amplitude).

Histopathological analysis was performed by fixing the samples in a 10% formaldehyde solution, then decalcifying them by keeping them for 2 weeks in Histo-Decal (Histo-Decal, Pantigliate, Italy). Subsequently, the samples were embedded in paraffin (Histopar—histological paraffin with 5–7% natural wax, Remed ProdImpex, Bucharest, Romania) then sectioned serially (thickness/section: max 5 µm) and finally stained with hematoxylin–eosin. All histological slides thus obtained were examined blindly under a light microscope (DM 4000B, Leica, Wetzlar, Germany) by a specialist histopathologist.

### 2.8. Statistical Analysis

The sample size was calculated by G*Power 3.1 (Düsseldorf, Germany) where error α = 0.05%, and a power of 80% was set. The number of animals in this study was set to 30, taking into account the complex steps of the experiment, the severity of the applied procedures, and the duration of the study. The normal distribution of data was analyzed using the Shapiro–Wilk test after which we performed a statistical analysis (Version 9.4.1, GraphPad Prism Software Inc., La Jolla, CA, USA) of the data applying a one-way ANOVA test and Bonferroni test with multiple comparisons between group C and group AAi and group C and group BL. Statistical significance was established for *p* < 0.05.

## 3. Results

**Figure 2:**
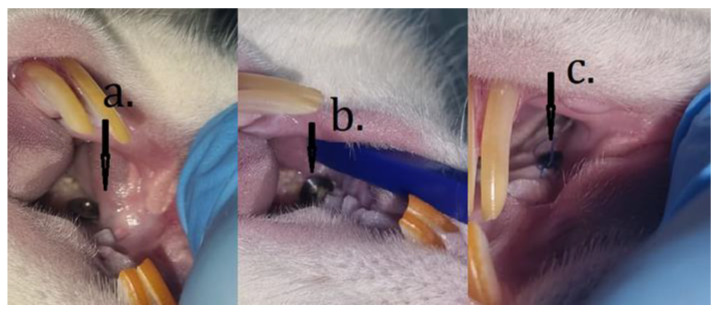
Implant appearance at the end of the contamination period with *A. actinomycetemcomitans*, *S. oralis*, and *F. nucleatum* where the position of the implant above the gingival margin (**a**–**c**), attached dental plaque (**b**), can be seen.

Specific clinical signs of PI were observed starting from the second week of oral contamination when the implants appeared above the gingival margin, and later, they were covered with plaque, bleeding appeared on probing, and some of them became mobile (4–5 weeks after bacterial contamination), as shown in Figure 2.

**Figure 3:**
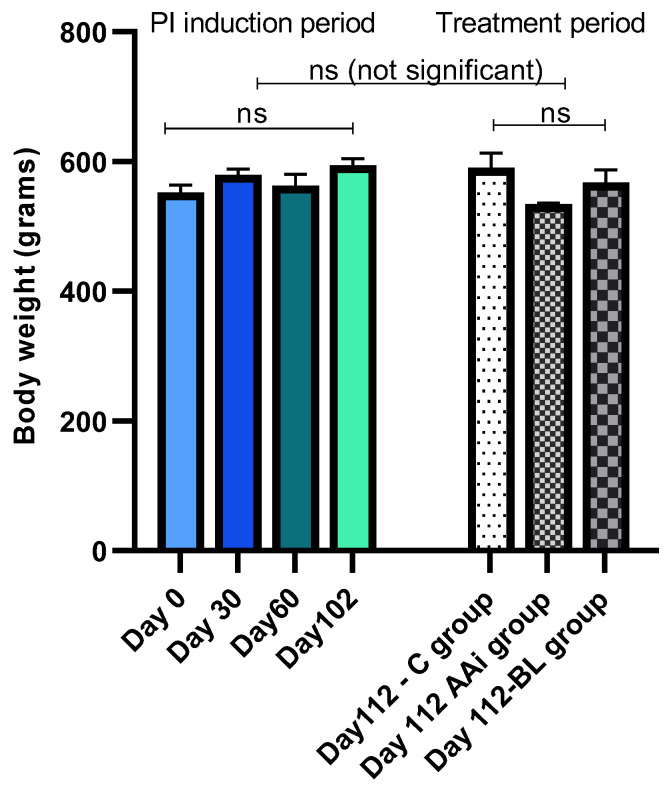
The evolution of body weight during the PI induction period (day 0–day 102) and after treatment/batch administration (day 112).

During the treatment period, the animals showed no signs of discomfort. Body weight did not change significantly in any group, and only group AAi showed more pronounced but not statistically significant decreases in body weight compared to groups C or BL (Figure 3). From a clinical point of view, no particular local signs were observed.

The implant survival rate (number of final residual implants) in each group was recorded at the end of the study. The initial number of implants fitted was 10 for each experimental group. The number of implants and survival rates per group are shown in Table 1. The implant survival rate in the control group was 0%, 20% for the AAi group, and 16% for the BL group.

**Figure 4:**
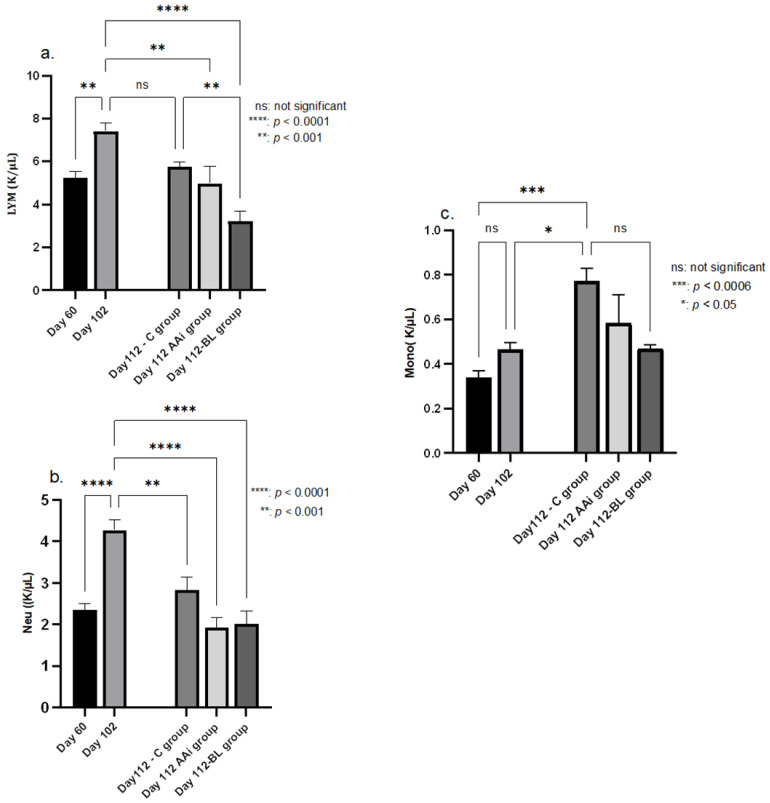
Lymphocyte (L YM—(**a**)), neutrophil (Neu—(**b**)), and monocyte (Mono—(**c**)) levels in response to the treatments administered compared to the onset (day 60) and end of the oral contamination period (day 102) when PI was in full progression. Once treatment was initiated, neutrophils and lymphocytes in the AAi and BL groups were significantly lower (*p* < 0.0001) than on day 102 when PI was clinically active. Monocytes were the only elements whose values continued to increase (*p* < 0.05) in group C, compared to the antibiotic- and bacterial lysate-treated groups whose values remained close to those of the induction phase. Only group BL recorded lower monocyte values compared to day 102 but without statistical significance.

The hematological examination of all treated groups (regardless of the solution administered) showed low levels of lymphocytes and neutrophils compared to day 102, the day that corresponded to the end of oral contamination, with a *p*-value between 0.001 (lymphocyte levels in the AAi group) and 0.0001 (lymphocytes and neutrophils in the BL group). Monocytes were the only elements whose level remained on an upward trend in group C compared to day 102 (*p* < 0.05); the other two groups did not show statistically significant values. It should be noted that the BL group reacted more markedly in terms of the reduction in the number of monocytes in the circulating blood than the AAi group, as can be seen in Figure 4.

**Figure 5:**
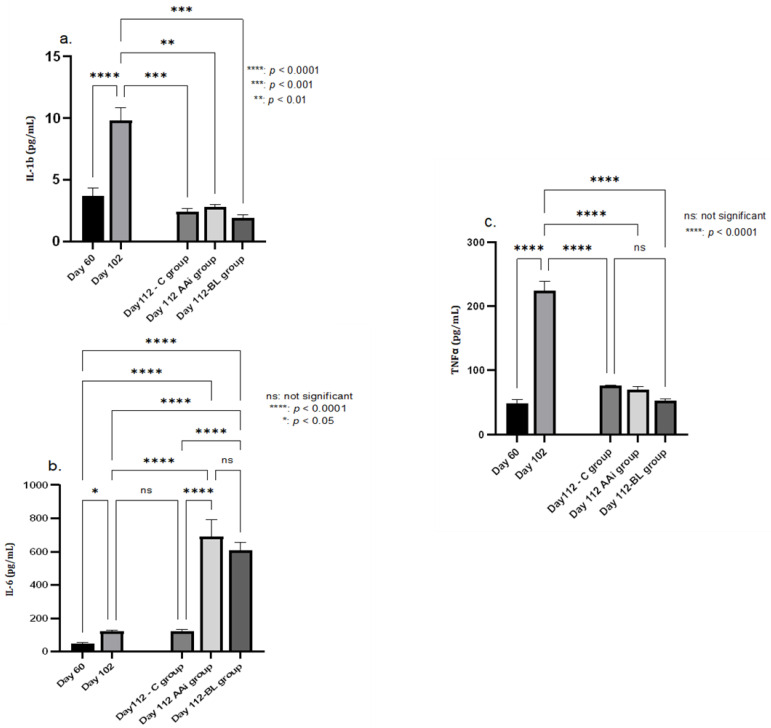
The evolution of proinflammatory cytokines IL-1b (**a**), IL-6 (**b**), and TNF-⍺ (**c**) following treatments compared to day 102. IL-1b levels decreased in all groups upon treatment initiation, with considerable statistical significance in both the AAi (*p* < 0.001) and BL (*p* < 0.001) groups. IL-6 showed a very high level in the AAi and BL groups (*p* < 0001) compared to day 102, and TNF had a similar evolution as IL-1b, with a decrease in all groups (*p* < 0001) compared to the induction phase of the disease.

Following the analysis of the main proinflammatory cytokines in PI, we could observe a significant reduction in all groups in terms of IL-1b (*p* < 0.01, AAi group, *p* < 0.001 in BL group) and TNF-⍺ (*p* < 0.0001 in BL group) levels compared to day 102. Related to IL-6, the groups that recorded values with strong statistical significance were the AAi- and BL-treated groups where *p* < 0.0001, compared to day 102. In an analysis of cytokines during the treatment period, the AAi and BL groups showed an accelerated increase in IL-6 (*p* < 0.0001) compared to the C group, with IL-1b and TNF-⍺ registering close values between groups without statistical significance (Figure 5).

**Figure 6:**
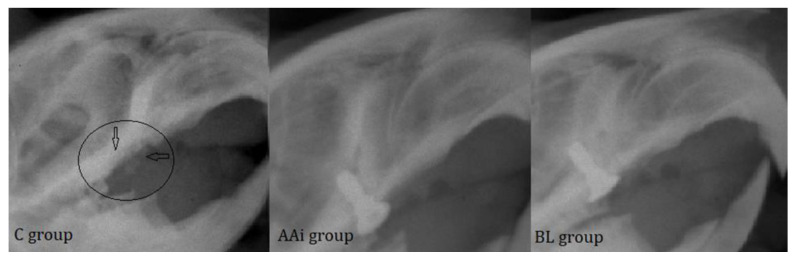
The radiological appearance of bone support after implant loss where resorbed bone can be seen, with irregular edges (circle and black arrows) in C group and implants in groups AAi and BL at the end of the study.

Dental X-rays are an integral part of the assessment of peri-implant disease for patients with clinical evidence of tissue destruction around teeth and/or implants. A careful review of the current approach to PI diagnosis shows that radiographs inform only a small proportion of the condition. The area in peri-implant assessment where radiographs play a key role, however, is in treatment planning, making it possible to observe outcomes after curative measures have been instituted. Following radiological examination, in the control groups, where we did not find the implanted devices, we could observe a high level of bone resorption, with a widened aspect of the bone socket (Figure 6, black arrows). In animals treated with antibiotics or bacterial lysate, the implants showed a correct position in the bone and optimal osseointegration (Figure 6).

The histological sections were made from samples of implanted jawbone after implant removal (groups AAi and BL) or samples of bone remaining after implant loss (group C). As in a previous study [34] and adapting the results to the lesion classification system proposed by Bleich et al. [35], we quantified the lesions detected, depending on the treatment of each group by being interested in the host immune response in peri-implant tissues, giving scores ranging from 0 to 4 (no histologically severable detectable elements–severe) which we later summarized and represented in Table 2.

**Figure 7:**
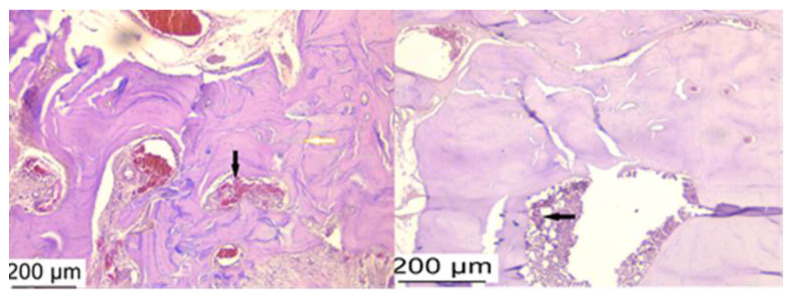
Histological appearance at the level of the implanted bone: the disorganization of the bone plates (yellow arrow) accompanied by hemorrhagic and inflammatory infiltrate (black arrow) observed in the control group. Hematoxylin–eosin staining, ob 10×.

**Figure 8:**
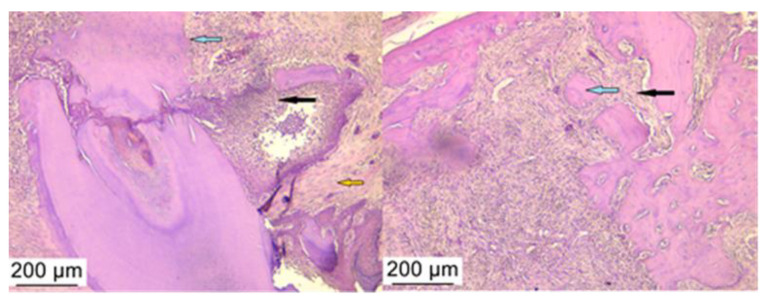
The histological aspect found in the group with PI treated with antibiotic and anti-inflammatory drugs where the persistence of the inflammatory infiltrate rich in lymphocytes and neutrophils (black arrow), fibrous infiltrate (orange arrow), and advanced process of bone destruction (blue arrow) can be observed. Hematoxylin–eosin staining, ob 10×.

**Figure 9:**
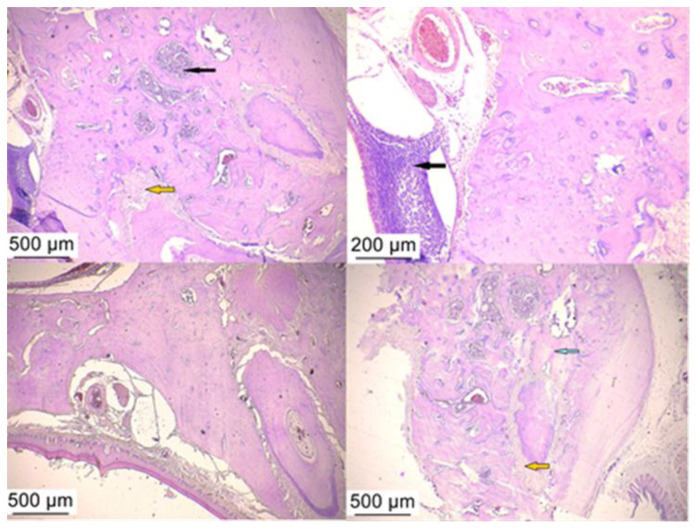
The histological aspect observed in the group with PI treated with bacterial lysate where the inflammatory infiltrate is in a moderate amount (black arrow), the bone destruction (blue arrow) being compensated by fibrous tissue agglomerates (orange arrow). Hematoxylin–eosin staining, ob 4×, and 10×.

A histological examination of hematoxylin–eosin-stained slides assessed the presence of inflammation in peri-implant tissue after treatment with bacterial lysates, antibiotics, and anti-inflammatories. The control group showed the presence of a junctional epithelium impregnated with hemorrhagic areas and neutrophils but also rich fibrous tissue (Figure 7). More than half of the samples from the PI groups (regardless of treatment) showed the infiltration of inflammatory cells in the peri-implant tissue, including neutrophils, lymphocytes, and plasma cells, more pronounced in all AAi (Figure 8), moderate in the BL group. Also, in the latter group (BL), in addition to the migration of inflammatory cells, an increase in the number and thickness of collagen fibers in the surrounding soft tissue was observed (Figure 9).

**Figure 10:**
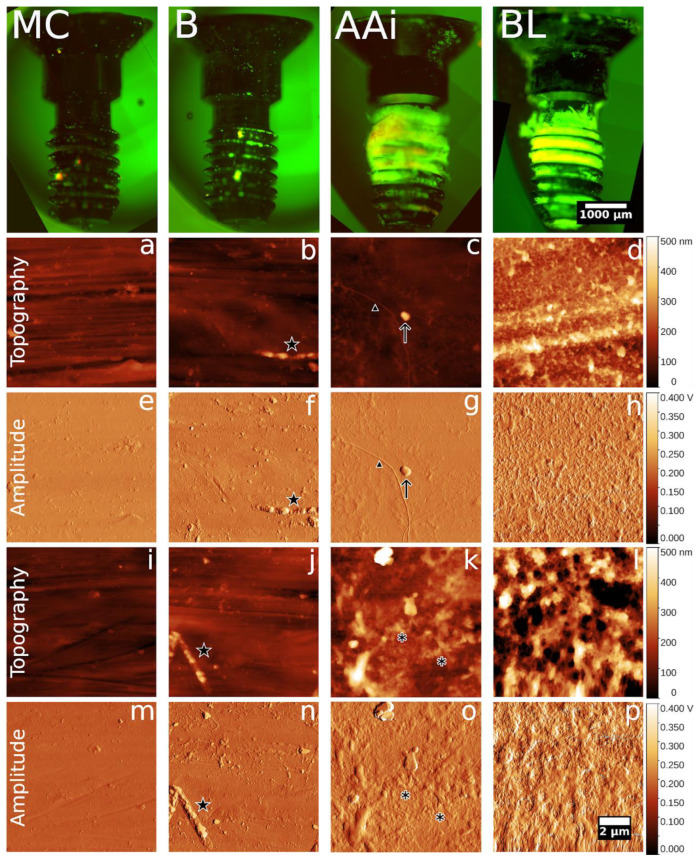
Fluorescence and atomic force microscopy analysis of surviving implants retrieved from the in vivo experiments and in vitro bacteria colonized controls; top—composite fluorescent micrographs of DAPI (green) and EOSIN (red)-stained implants, reconstructed from approximately 35 tiles per implant, (**MC**)—in vitro media control, (**B**)—in vitro bacterial colonization, (**AAi**)—ex vivo from AAi group, (**BL**)—ex vivo from BL group. Bottom—representative AFM scans (2 per condition) corresponding to top panel, (**a**–**d**,**i**–**l**)—topography images false colored from 0 to 500 nm, (**e**–**h**,**m**–**p**) amplitude images false colored from 0.000-0.400 V. Star—*F. nucleatum*, arrow—potential bacteria, asterisk—potential clusters of *A. actinomycetemcomitans*, triangle—collagen fiber.

Fluorescence microscopy showed that a high amount of tissue and biological fouling was maintained on the implant surface after the fixation and extraction protocol (Figure 10, top). Higher magnification investigations (20×, Supplementary Figure S1) of select areas revealed that most of the fluorescent signal was due to bone tissue autofluorescence, the extracellular matrix (eosin stain), and cell nuclei (DAPI). No structures indicative of bacterial biofilms were identified even at this higher magnification on the ex vivo samples. On the in vitro inoculated control (Figure 10B), a weak network of nucleic acid staining was evident when compared to the culture media-only control (Figure 10MC). Consequently, multiple 10 × 10 µm AFM scans were performed at random locations on different areas of the implants. Representative images for the upper shaft (a–h) and thread area (k,l,o,p) are presented in Figure 10. No scans could be performed in the thread area of in vitro control implants since the high slope, in absence of biological tissue, impeded proper scanning.

Implants retrieved from the AAi and BL groups frequently showed incipient bone-like mineral deposits (Figure 10c,d,g,h), well-developed trabecular bone (l,p), and collagen fibers (triangle in c,g). On a very limited number of scans, all from the AAi group, structures with bacterial morphology could be observed, but these events were too rare to ascertain any difference between samples or support any conclusion of widespread bacterial colonization. Clusters with morphology indicative of *A. actinomycetemcomitans* (asterisk in Figure 10k) were only observed in a single scan of the implant retrieved from the AAi group, embedded in the bone structure adhered to the thread area. On the in vitro colonized control implant, most scans showed bacteria with the characteristic morphology of *F. nucleatum* adhered on the surface.

## 4. Discussion

In experimental research, rats are often used because of their advantages of size, reproduction rapid growth cycle, maintenance, and easy handling. In terms of bone structure, rats are very similar to humans although bone volume shows variations according to gender, age, weight, and sex [36]. Thus, bone density tends to increase as animals age, and hormonal variations also influenced a study involving the use of bone support [37,38]. Therefore, for this study, rats aged 20 weeks were used, based on these considerations and especially on the fact that after this age, the animals are considered old, thus wishing to simulate a situation similar to humans in which the need for an implant is greater in the second half of life. In the establishment of the PI model, some difficulties were encountered concerning the oral opening or the fragility of the molar roots. Rat first molars have three buccal and two palatal roots, and the instrumentation and technique adapted to the size of the animal allowed for the extraction of the premolars without a great risk of root fracture. Many studies using small animal models for implant insertion have used modified instrumentation to achieve the desired results [39]. The implant site was the extraction site, and the execution of the maneuver was challenging in many ways. The first challenge was the limited space, the second was the increased risk of the perforation of the jawbone, and the third was the stability of the implant. To achieve good stability, it was necessary to reduce movement at the implant–bone interface, thus avoiding the degradation of the newly formed alveolar bone post-extraction. For implant mounting, the palatal alveolus was chosen because of its thickness suitable for the implant size, and by doing so, it was ensured that the bone support is a suitable one that promotes osseointegration, as some authors showed in a meta-analysis [40].

Microbiological diagnosis has been proposed as a possible approach to detecting the most aggressive periodontal pathogens [41]. Since in some patients, there is no clear difference related to the bacterial species involved in peri-implant health and disease, we resorted to oral contamination with common peri-implantitis strains, namely *A. actinomycetemcomitans*, *S. oralis*, and *F. nucleatum*. The bacterial species identified in PI are predominantly anaerobic, Gram-negative, and not related to a uniform microbial profile as found in periodontitis [42]. A significant difference was not observed between the strains isolated from the PD site and PI [43]; therefore, we chose PI-specific bacterial strains. In a healthy organism, the oral bacterial community is in a state of equilibrium with the host, but the intervention of risk factors causes biofilm formation and the implicit occurrence of inflammation. Consequently, the increase in the number of strains adhering to the initial biofilm results in difficult therapeutic management [44]. Thus, PI treatment aims to control infection and reduce bacterial substrate.

Clinically, no differences were observed between the antibiotic-treated and bacterial lysate-treated groups of animals, but after the therapeutic approach, the implant survival rate was higher in the bacterial lysate-treated groups and 0 in group C. For the fact that no device was found in the control group, we can hypothesize that PI still progressed in these animals, speeding up the implant removal process. From a general perspective, out of 30 implants initially fitted, at the end of the study, almost half were still found, which is in line with a recent meta-analysis [45] where out of 213 implants, only 163 survived on the rat model with ligation- or bacterial contamination-induced PI. Recommendations for clinical changes in peri-implant tissues refer to immediate debridement, oral washes with disinfectant substances, and systemic antibiotic therapy, but in animals, the use of antibiotics may influence the healing process and immune response [46]. The results of this study encourage further investigation into the efficacy of bacterial lysates in the healing of PI and their use instead of antibiotics, especially due to the similarity of the response of the animal body to the administered treatments, demonstrated by the reduction in the inflammatory response observed both hematologically and especially histopathologically. In the available literature, bacterial lysates, it appears, may be an option for patients with recurrent respiratory infections, allergies, atopic dermatitis, or asthma [47]. They are antigens obtained from the inactivation of pathogenic bacteria that once had immunogenic properties. The route of administration of bacterial lysates influences their immunogenic capacity in that they exert their action on dendritic cells whose predominant distribution is in the upper respiratory tract and oral cavity [48]. Bacterial lysate preparations in the form of sublingual tablets or oral solutions have maximum efficacy in the oral cavity without the need for antibiotic pharmacokinetics, and therefore, oral diseases such as PI can be treated with such products.

Based on the current knowledge that PI and PD have similar infectious etiology, the use of systemic antibiotic therapy is considered by some authors to be standard [49], even though in recent years, the increasing antibiotic resistance of pathogenic and opportunistic bacteria has developed. For this reason, it is imperative to develop new therapies that circumvent the unwarranted use of antibiotics. Saline is the most widely used in the care and treatment of oral diseases [50], and the present study compared the effect of antibiotic therapy and bacterial lysate with the effect obtained after saline lavage.

For the assessment of inflammation status, cytokines provided valuable information. The role of IL-1b (a basic cytokine in the inflammatory response) is a major one in the pathogenesis of PI, and an increased level indicates soft tissue inflammation and the degree of bone resorption. IL-1b correlates positively with implant failure [51], even being a predictor of the degree of severity of PI [52]. This cytokine shows higher secretion in the early phases of the disease but not as high as found in peri-implant mucositis [53,54].

In PI, TNFα occurs at a high level in both soft and hard tissues and is directly proportional to the degree of bone destruction and, in turn, may be a predictor of PI severity [51]. Some studies show that this cytokine could be used in the diagnosis of PI which would involve the use of standard antigen swabs with which to collect saliva from patients with suspected disease [55].

IL-6 is excreted by osteocytes and plays a role in osteoclast formation. By some specific mechanisms, IL-6 can block the effects of TNF-alpha. In bacterial-induced PI, IL-6 is secreted by macrophages and has a proinflammatory role, being found in significant amounts in the sulcular fluid [56]. Increased IL-6 indicates the onset and progression of PI, and instituting treatment does not ensure the control of the cytokine [57]. The same aspect could be observed in our experiment where in the AAi and BL groups, IL-6 levels remained elevated compared to the control group. Moreover, some studies claim that a high cytokine concentration could protect the body against IL-1b- and TNF-⍺-induced osteoclastic re-obstruction [58]. To decrease the average concentration of IL-1b and TNFα, the primary role is played by the correct peri-implant therapy to maintain these cytokines in normal parameters. A study by Gomes et al. showed that instituting peri-implant therapy without proper hygiene maintenance has unfavorable results over time [59]. Even disinfectants (chlorhexidine) are not highly effective in treating oral diseases, and in addition to their action, ozonized gels have proven superior as an alternative treatment [60]. The obtained results in the present study reinforce the theory that the applied treatments have favorable effects by demonstrating the reduction in IL-1b and TNF-α in both the AAi and BL groups; in this situation, bacterial lysates can be considered fundamental components of therapeutic tactics similar to antibiotics. The cytokine response to bacterial lysate action requires further investigation because few studies have addressed this therapeutic method in PI, with no evidence reported in the literature. However, a periodic analysis of cytokine assays facilitates the diagnosis of PI.

It is also required to investigate the osteoimmunological mechanisms involved in alveolar bone shaping. The complex of proteins responding to lesion recognition receptors leads to the secretion and maturation of proinflammatory cytokines along with the activation of the inflammatory response. Inappropriate activity of the protein complex may allow for the persistence of infection and uncontrolled resorption of alveolar bone in PI through the regulatory mechanisms of osteoclasts, osteoblasts, osteocytes, macrophages, monocytes, neutrophils, and adaptive immune cells [61]. Osteoclasts originate from monocytes which are initially involved in implant osseointegration, implant stability, and the long-term maintenance of the marginal bone level. The obtained results on monocyte response confirm this hypothesis by the fact that they showed increased reactivity, especially in the control group, and by the fact that in the AAi and BL groups, monocyte levels decreased, indicating the efficacy of the treatments in implant stability. The association between PI and the increase in neutrophil count is due to the interaction between the infectious agent and the host immune cells which results in the massive production of leukocytes [62]. In PI induced by *A. actinomycetemcomitans*, *S. oralis*, and *F. nucleatum*, this could be observed as well as their attenuation following the institution of treatments.

The application of both treatments for PI resulted in the compensation of bacterial dysbiosis and peri-implant tissue recovery. The 10-day treatment period was sufficient to evaluate the effects, especially considering the rapid growth rate of these animals and the fact that, at adult age, each day of the animal is approximately equivalent to 34.8 human days [63], so in the case of the translation of the treatment method, it would be recommended that bacterial lysates be administered for longer periods of time. Bacterial lysates can be considered a strong competitor for antibiotic therapy, a fact that was evident from the histologically observed lesional picture, where the inflammatory infiltrate was less pronounced, as was the degree of bone destruction. Histopathologically, the lesions detected confirmed the attenuation of the inflammatory process in the peri-implant sac, regardless of the treatment applied (antibiotic or bacterial lysate). Only in antibiotic-treated animals did the inflammatory infiltrate remains increase in the samples analyzed. As suggested by other authors, experimental PI studies should be continued to establish the mechanisms of tissue development, protection, and recovery under pharmacological correction by bacterial lysate [64,65].

Radiological examination, as an integral part of PI diagnosis, helped establish implant integrity throughout this study. Through it, the level of bone density was compared, and in the control groups, this density was much lower (also revealed by the degree of survival of the implants) than in the antibiotic- and bacterial lysate-treated groups. An analysis of the obtained images revealed optimal osseointegration in the implants and bone densities characteristic of reduced inflammatory processes.

An AFM examination of extracted implants revealed mostly regular bone tissue structures, supporting the rest of the data showing proper osseointegration in both treated groups. AFM imaging is able to readily allow for the assessment of bacterial colonization and measurement of biofilm properties on in vitro colonized implants from different materials [66], as also shown in the current study. While probable planktonic bacteria and bacterial clusters were identified on ex vivo implants from the AAi group alone, these observations were too rare to conclude any difference between samples or the widespread colonization of surviving implants at the assessed timepoint. A major limitation in this respect is the premature loss of all implants in the in vivo control group which might have allowed for a comparison of bacterial biofilm formation at higher levels of bioburden.

However, although the results of this study confirm the null hypothesis which was based on the similar response of the lysates to antibiotic therapy in fighting the bacterial agents involved in PI, there are some limitations to mention. One of them is related to the oral flora of the rats which is quite consistent and could have influenced the results of this study. A well-established and reproducible PI model is extremely important when testing new therapies. The induction time of the disease (6 weeks) is short compared to the clinical situation in which PI evolves in a few years, and the fact that in the control group, there were no implants to be analyzed compared to the treatments applied to the AAi and BL groups may mean that the disease evolves even in the absence of the administration of bacteria (which would be similar to humans) but which may raise doubts about the efficacy of the lysates or even antibiotics on the model created. Given the fact that the treatment of PI induced by three bacterial strains responsible for the disease in humans has been attempted, another limitation relates to the efficacy of the lysates on the microbial complexity of human PI patients, and the translation of the method would be interesting to follow, especially if such lysates were made from the specific pathogenic flora of each patient or a complex bacterial lysate comprising a suite of the most pathogenic oral bacteria.

New therapies can be tested on the PI model created, but further research is needed on the efficacy of bacterial lysates on this disease, both in vitro and in vivo, perhaps using other bacterial strains (to create the bacterial lysate) or other methods of disease induction (without bacterial involvement), as mentioned above. An important criterion in evaluating the efficacy of lysates refers to immunological analysis, and in future studies, the mechanism of action of lysates in triggering the immune response should be further investigated. The bacterial lysate therapy of PI is a new topic, and the results presented are promising for further research and especially for the development of specific PI therapies.

## 5. Conclusions

The research in this study aimed to evaluate the effect of antibiotic therapy and bacterial lysate treatment on peri-implantitis. By corroborating the results of hematological, immunological, radiological, and histopathological analyses and implant survival rates, we can state that the treatment with bacterial lysate had a similar effect to the standard treatment, based on antibiotics and anti-inflammatory drugs when compared to untreated controls. Furthermore, slightly higher implant survival rates were observed in the bacterial lysate-treated group compared to the group receiving antibiotics. Bacterial lysate administration is a valid treatment option developed for PI because of pathogenic bacteria, and a personalized bacterial lysate-based therapy could reduce unnecessary antibiotic consumption and thus contribute to reducing antibiotic resistance. Furthermore, bacterial lysates, as well as other disinfectants or substances with disinfecting or reparative properties (e.g., ozone-based gels), can be considered valuable alternatives for the treatment of peri-implantitis.

## Figures and Tables

**Table 1:** Number of implants and implant survival rates/group.

	C Group	AAi Group	BL Group
Number of animals	10	10	10
Number of implants	10	10	10
Final implants	0	6	8
Survival rate of implants	0%	60%	80%

**Table 2:** Semi-quantitative scoring for histological evaluation in groups of animals undergoing treatment.

Parameter	Score	Occurrence of Injuries	Group
Inflammatory infiltrate in the peri-implant pocket	0	No neutrophils	-
	1	Rare neutrophils	AAi group BL group
	2	Neutrophilia	C group
Fibrosis	0	Absent	-
	1	Mild	-
	2	Moderate	AAi group BL group
	3	Severe	-
Bone–implant interface	0	Completely cured	AAi group BL group
	1	Rare osteoclasts	AAi group BL group
	2	Mild osteoclasts	-
	3	Moderate osteoclast activity	C group
	4	Severe osteoclast activity	-

## Data Availability

The original contributions presented in the study are included in the article, further inquiries can be directed to the corresponding authors.

## Supplementary Materials

The following supporting information can be downloaded at: https://www.mdpi.com/article/10.3390/microorganisms12081537/s1, Figure S1: Representative 20× magnification fluorescence images of retrieved implant showing DAPI stained cell nuclei, Eosin stained extracellular matrix and bone tissue autofluorescence/nonspeciffic staining.
