# Supplementary material for: A Comparative Analysis of the Efficacy of Bacterial Lysate versus Antibiotic Therapy in the Treatment of Experimental Peri-Implantitis in Rats
Source: Microorganisms. 2024 Jul 27;12(8):1537. doi: 10.3390/microorganisms12081537 (PMC11356466; doi:10.3390/microorganisms12081537)
Supplement: Supplementary file 1 [file microorganisms-12-01537-s001.zip › microorganisms-3103120-supplementary.pdf]

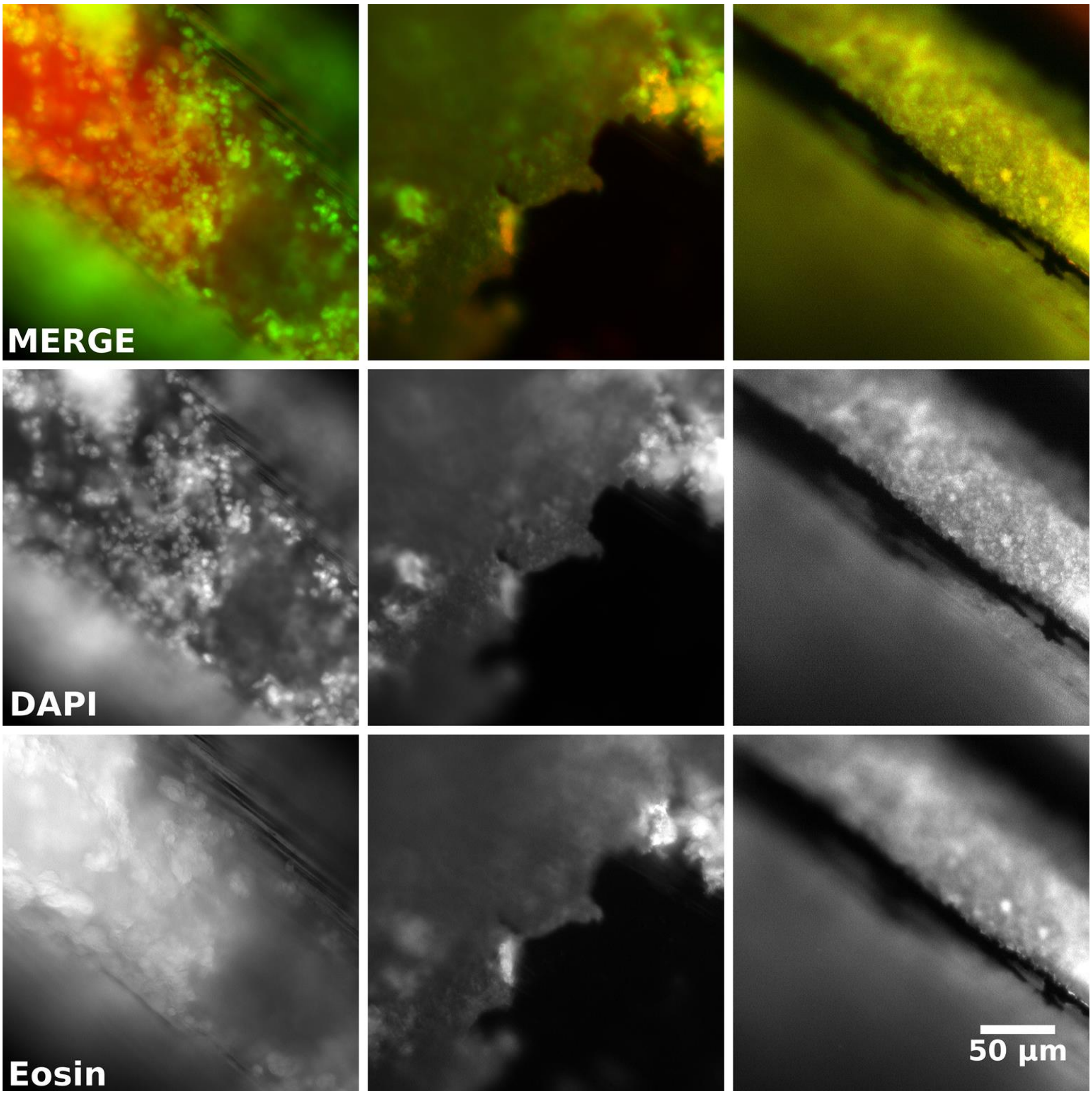

Figure S1. Representative 20× magnification fluorescence images of retrieved implant showing DAPI stained cell nuclei, Eosin stained extracellular matrix and bone tissue autofluorescence / nonspeciffic staining.
